# Computer and Internet Use Among Children and Adolescents in Greece: Levels of Addiction

**DOI:** 10.7759/cureus.75942

**Published:** 2024-12-18

**Authors:** Aikaterini Toska, Konstantinos Karampelas, Evangelos C Fradelos, Pavlos Sarafis, Ioanna V Papathanasiou, Ioannis Koutelekos, Konstantinos T Petsios, Constantinos Togas, Angela Notou, Maria Saridi

**Affiliations:** 1 Epidemiology and Public Health, University of Thessaly, Lamia, GRC; 2 Social and Educational Policy, University of Peloponnese, Corinth, GRC; 3 Nursing, University of Thessaly, Larissa, GRC; 4 Nursing, University of West Attica, Athens, GRC; 5 Nursing, National and Kapodistrian University, Athens, GRC; 6 Psychology, Panteion University, Athens, GRC

**Keywords:** addiction, adolescents, children, computer, greece, internet, parents

## Abstract

Objective: The digitalization of our daily living has changed dramatically the internet and digital media use among all ages. However, children and adolescents are particularly vulnerable to potential dangers and addiction risks. Our objective was to evaluate the computer and internet addiction levels in children and adolescents through their parents’ reports.

Methods: A cross-sectional study was conducted in the region of Megara (Attica, Greece) for two months (September-November 2022). In total, 205 parents (74 fathers, 131 mothers) of school-age children participated, completing the study questionnaire (demographic data and the Adolescent Computer Addiction Scale - parental version (20 items in a 1-5 Likert Scale).

Results: Based on the addiction scale score, only 2.45% (N=5) of the parents rated high scores indicating serious addictive behaviors regarding internet use. The majority were parents of boys (58%) and the mean age of children was 10.92±2.81years (five to 18 years). According to parental reports, the mean age of primary access to a computer or device with internet access was 7.18±2.29 years (two to 13 years). Regarding internet use, 58.5% of the children had access to the internet via smartphone, used the internet one to two hours/day (46.1%), and 62% of children had internet access in their bedroom. Children whose parents used the internet more than five hours/day displayed a greater addiction (F=3.06, df=3.187, p=0.03). A significant positive correlation was also found among children age (r=0.388, p<0.01), computer and internet availability in children’s bedrooms, and internet addiction (t=4.43, df=189, p≤0.001 and t=3.88, df=189, p≤0.001, respectively). In summary, parents reported moderate or severe addiction in 7.2% of boys and 5.1% of girls. However, it should be noted that the exact cut-off points were difficult to determine due to a lack of objective criteria for diagnosing addiction severity.

Conclusions: The levels of addiction in our study according to the parents' reports were limited and only a small number of parents reported highly addictive behaviors. However, there is a direct correlation between increased parental internet use that can significantly increase children's addiction levels. Parents act as role models that may increase children’s exposure to the internet and lead to addiction.

## Introduction

In the modern world, the use of digital devices and the internet is particularly widespread and is increasing at a rapid rate, especially after the COVID-19 pandemic, among all age groups [1-3]. Children and adolescents, in particular, use the internet as their primary means of communication and socialization [4]. This new reality inevitably raises significant concerns about the actual benefits of technology use among children, especially preschoolers. There is also growing concern about the risk of addiction and other issues, such as internet-related psychosocial disorders, exposure to inappropriate or unsafe content, and compromised privacy and confidentiality [5]. It is widely acknowledged that internet addiction leads to significant disturbances in all areas of human functioning [6].

Recent research data indicates increased rates of unrestricted internet use by children, usually with limited or without parental supervision [7-9]. Therefore, parents must deal with the excessive time their children spend on screens as well as their children's resistance to reducing the time of use which often leads them to neglect other productive activities, sports, or even schoolwork. Therefore, children and young adolescents are at higher risk of developing an internet addiction disorder and engaging in problematic internet use, in comparison to other age groups [10,11]. Boys show greater addiction to online games and girls tend to be addicted more to the use of social networks [12]. Public health professionals and psychologists have pointed out the importance of preventive measures regarding internet addiction and the assessment of internet-related disorders, which are now considered to belong to behavioral addiction [13].

A significant similarity between internet addiction and addiction to various substances has been documented. In particular, the use of devices to access social media such as smartphones affects the production of dopamine, a chemical associated with addiction to smoking, alcohol, and gambling [14]. Children who use these devices often show a similar pattern of addiction and use the internet to relieve stress levels caused by any trigger or stressful event, which leads them to resort to digital devices, experiencing a similar state of substance addiction. Tablets, laptops, and smartphones provide unlimited internet access to youths, even preschoolers. Parental control, parental awareness, and parental internet habits are of crucial importance and affect children’s exposure to addiction. Parents should set rules and monitor the use of devices with internet access as the basis for the prevention of internet addiction. Given that the phenomenon of internet addiction is a significant problem that affects the pediatric population worldwide as well as in Greece, the main aim of this study was to evaluate the level of internet addiction in children and adolescents through their parents' reports [8].

## Materials and methods

The purpose of the research was to assess children's addiction to computer devices and internet use through their parents' reports. The research employed a cross-sectional design and was conducted from September to November 2022 on a convenience sample of parents in a provincial city in the Peloponnese region of Greece. Originally 300 questionnaires were distributed to parents of school children attending public schools in the region of Megara. In total, 205 parents (74 males, 131 females) returned the questionnaire and were included in the study (response rate=68.3%).

The study inclusion criteria were set as follows: a) the parents (men or women) have at least one child of school age, b) they had signed the informed consent to participate, and c) they were able to speak and understand the Greek language. Both parents of the same child could not participate, or if they did, they were required to focus on different children. Exclusion criteria were set as follows: a) parents who were not able to speak and understand the Greek language and b) parents who failed to provide complete information in the questionnaire. 

The questionnaires were distributed by the researcher and completed in printed form. Participants were allowed to take them home, fill them out, and return them in the following days.

The study questionnaire included three separate parts: a) parental demographic information, parental computer devices, and internet use; b) child demographic information, child’s computer devices, and internet use; and C) the Greek version of the parental form of the Adolescent Computer Addiction Scale [15]. The scale consists of 20 questions designed to assess children’s and adolescents’ addiction to computers and the internet, as perceived and evaluated by their parents. An example question is "How often do you find that your child neglects homework in order to spend more time on the computer?" Questions are answered using a five-point Likert scale (1=not at all, 5=always), with the total score ranging from 20 to 100 points. Higher scores indicate a greater level of addiction to computers and the internet, as perceived by the parent. The scale has demonstrated excellent psychometric properties.

Data analysis was performed using SPSS v. 26.0 (Statistical Package for the Social Sciences, Chicago, Illinois). Categorical variables are presented as frequencies and percentages. Continuous variables were expressed as mean and standard deviation (SD). The Kolmogorov-Smirnov test was used for evaluating the normality of the data distribution. A significance level was accepted as 0.05.

Ethical considerations

For the conduction of the survey, prior approval was received from the General Assembly of the Department of Social and Educational Policy (223rd/06.10.2022 item number 16.1). The parents were fully informed about the study's aim and methodology. An informed consent form was provided to them before the questionnaire completion. They were assured that their responses would be anonymous, and their answers would be used solely for research purposes. Furthermore, they were informed that their participation was voluntary and that they had the right to withdraw their consent at any time, without the need to give further explanations.

## Results

Demographic characteristics of participants (parents) and internet use habits

Most participants were married (Ν=167, 81.9%), were high school graduates 43.6% (Ν=89), and had two children (57.6%). Table 1 presents in detail the participants' demographic data.

**Table 1:** Participants demographic data

Parent gender	Frequency	Percentage, %
Male	74	36.1
Female	131	63.9
Total	205	100
Marital status		
Single	12	5.9
Married	167	81.9
Divorced	19	9.3
Widowed	6	2.9
Total	204	100
Education level		
Elementary school	23	11.3
Gymnasium	18	8.8
Lyceum	89	43.6
Student	7	3.4
University/technological institution	51	25.0
MSc/PhD	16	7.9
Total	204	100
Professional status		
Household	38	18.5
Unemployed	19	9.3
Worker	15	7.3
Farmer	15	7.3
Public/state employee	32	15.6
Private employee	46	22.4
Freelance worker	40	19.5
Total	204	100
Number of children		
One	34	16.6
Two	118	57.6
Three	37	18.0
Four	13	6.3
Five	2	1.0
Seven	1	0.5
Total	204	100
Number of boys		
None	38	18.5
One	99	48.3
Two	50	24.4
Three	16	7.8
Four	1	0.5
Five	1	0.5
Total	204	100
Number of girls		
None	56	27.5
One	107	52.5
Two	33	16.2
Three	5	2.5
Four	2	1.0
Five	1	0.5
Total	204	100%

Most parents (44.4%) stated two to three hours of internet use per day, while the vast majority (79%) had access to the internet via smartphone, followed by a desktop computer (39%), laptop (35.1%), or tablet (29.3%). Only in a few cases, internet access was available via internet café (0.5%) or otherwise (2.9%).

Most parents used the internet for information (64.9%) and for e-mail exchange (63.9%). A smaller percentage used it for other activities (Table 2). Almost two of three participants (62.9%, N=129) stated participation in informative lectures or discussions regarding the safe use of the internet.

**Table 2:** Parents' reasons for internet use (multiple-choice options)

Internet use	Frequency	Percentage, %
Professional reasons	95	46.3
Informative reasons	133	64.9
School homework	67	32.7
Games	48	23.4
Social nets (Facebook, Instagram, etc.)	123	60
Professional-scientific networking (Linkedin, Academia edu, etc.)	19	9.3
Watching movies/videos	72	35.1
Listening to music shows	57	27.8
Email exchange	131	63.9
Online purchases	100	48.8
Download music/videos	27	13.2
Other	1	0.5

Children’s demographic characteristics and internet use

The parents reported data regarding the demographic characteristics and the internet use of their child. They reported data for 119 (58%) boys and 86 (42%) girls, aged 10.92±2.81 years (five to 18).

Parents stated that more than six children in 10 (61%) were engaged in outdoor activities such as sports, gymnastics, dance, music, theater, ballet, and art for less than five hours per week. 

More than half of the children had access to a computer device with internet under the age of eight (mean age of first internet use was 7.18±2.29 years (ranging from two to 13 years old). Table 3 illustrates the age distribution of the child in his/her first experience of internet use.

**Table 3:** The child's age at first internet use

Age	Frequency (N)	%
2 years old	4	2.0
3 years old	1	0.5
4 years old	4	2
5 years old	15	7.4
6 years old	20	9.8
7 years old	25	12.3
8 years old	33	16.2
9 years old	29	14.2
10 years old	24	11.8
11 years old	28	13.7
12 years old	12	5.9
13 years old	7	3.4
Total	204	100

Almost one in two children have two to three hours of internet use daily. Noticeably, 25.5% of the children have internet access for more than four hours per day. Most children (N=120, 58.5%) use the internet via smartphone. Parents stated that only a limited number of children would visit an internet café to have internet access and that was only for online gaming (N=7, 3.4%). Most of the children (62.4%) did not have access to a computer located in their bedroom.

Moreover, parents were asked to report computer device use without the internet. Noticeably, 56.6% of children did not use the computer without access to the internet.

Adolescent Computer Addiction Scale - parent edition findings

The reliability of the parental version of the Adolescent Computer Addiction Scale was excellent (Cronbach's α=0.944). Table 4 presents the mean score per item of the questionnaire. The complaints regarding time spent on computer devices (item 5) and asking for more time for its use (item 16) showed a higher mean score. On the contrary, the development of a new online friendship with other users showed the lowest score. The mean total score on the Adolescent Computer Addiction Scale - parent version was 46.07 (20-88). Based on the addiction scale total score, only 2.45% (N=5) of the parents rated high scores indicating serious addictive behaviors regarding internet use. Moreover, moderate or severe addiction was assessed in 7.2% of boys and 5.1% of girls. A great number of participants (N=84, 41.2%) stated a low total score (20-40). The rest had moderate scores that indicate a possible internet addiction that is difficult to determine due to the lack of specific score cut-offs. The exact cut-off points were difficult to determine since there are no objective criteria for diagnosing addiction severity.

**Table 4:** Mean score per question on the Adolescent Computer Addiction Scale - parent version

Items	Mean±SD	Min	Max	Range
1. How often do you find that your child stays connected to the computer (login) longer than he originally intended?	2.96±1.12	1	5	4
2. How often do you find that your child neglects his homework to spend more time connected to the computer?	2.72±1.16	1	5	4
3. How often do you find that your child prefers the thrill of being on the computer to contact with a person?	2.39±1.19	1	5	4
4. How often do you find your child forming new friendships with other computer users?	1.64±1.02	1	5	4
5. How often do you complain about the time your child spends on the computer?	3.16±1.27	1	5	4
6. How often do you find that your child's grades or school performance are negatively affected because of the time they spend on the computer?	2.27±1.22	1	5	4
7. How often do you find your child checking their messages before something else they should be doing?	2.36±1.42	1	5	4
8. How often do you find that your child's academic performance or productivity is negatively affected by their computer use?	2.31±1.22	1	5	4
9. How often do you find that your child tries to make excuses or lies when someone asks what exactly they are doing when they are connected to the computer?	1.92±1.11	1	5	4
10. How often do you find that your child pushes away unpleasant thoughts about his life by using reassuring thoughts related to computer use?	1.84±1.12	1	5	4
11. How often do you find that your child after previous use can't wait to get back on the computer?	2.77±1.10	1	5	4
12. How often do you think or your child tells you that life without the computer would be boring, empty, and joyless?	2.15±1.23	1	5	4
13. How often do you find that your child becomes abrupt, shouts, or reacts with irritation if someone disturbs them while connected to the computer?	2.49±1.29	1	5	4
14. How often do you find that your child loses sleep because of his late-night computer connection?	1.82±1.14	1	5	4
15. How often do you find that your child is concerned about connecting while away from the computer?	2.04±1.15	1	5	4
16. How often do you find your child saying "just a few more minutes" while logging on to the computer?	3.02±1.25	1	5	4
17. How often do you find that your child tries to limit the time he spends on the computer and fails?	2.20±1.20	1	5	4
18. How often do you find that your child tries to hide the time spent on the computer?	2.28±1.24	1	5	4
19. How often do you find that your child prefers to spend more time connected to the computer than going out with friends?	1.94±1.16	1	5	4
20. How often do you find that when your child is offline, they feel sad, moody, or nervous, which disappears when they are back online?	1.81±1.09	1	5	4

We investigated the possible correlation between children's gender in terms of computer and internet addiction, which showed that boys scored slightly higher than girls (47.53 vs. 44.10) on the Adolescent Computer Addiction Scale. However, this difference was not statistically significant (t=1.41, p>0.05). No significant correlation was also observed between parents' educational level and children's addiction (F=1.52, df=7,182, p>0.05).

**Figure 1:**
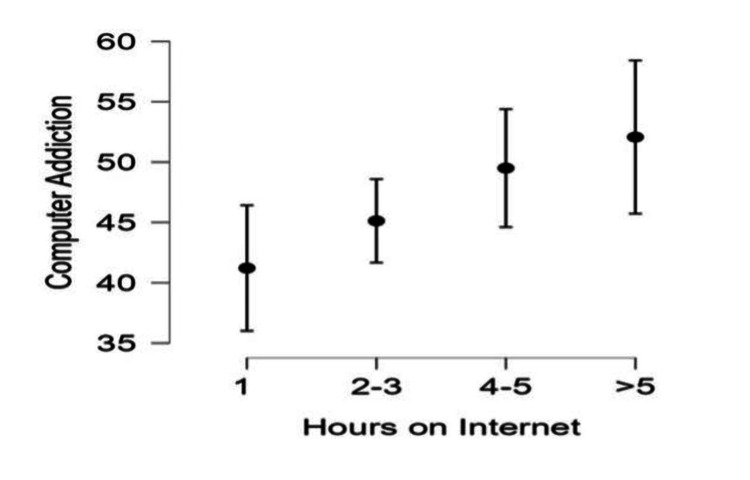
The levels of children's and adolescents' addiction to computers and the internet in relation to the frequency of internet use by parents

Regarding the correlation between children's and adolescents' addiction to computers and internet and the frequency of internet use by the parent, it was observed that the frequency of internet use by the parents had a significant effect on the addiction of children and adolescents to the use of computers and internet (F=3.06, df=3,187, p=0.03). The multiple comparison test (Bonferroni post hoc test) showed that children whose parents used the internet more than five hours/day had a higher score (M=52.07) than children whose parents used the internet for one hour/day (41.21) (Figure 1).

A positive correlation was noticed between adolescents' and children's computer and internet addiction and access to a computer in their room (M=52.55 for those who had and M=42.07 for those who did not) (t=4.43, df=189, p≤0.001). That was an interesting finding indicating a greater level of addiction in children who had access to a computer in their room in comparison to children who did not have such access. A significant correlation also emerged between children’s and adolescents’ addiction to computers and the internet and their access to the internet in their bedroom (M=49.53 for those who had and M=40.23 for those who did not) (t=3.88, df=189, p≤0.001). Specifically, children who had internet access in their bedrooms had a higher likelihood of addiction than children who did not have such access.

Finally, a significant positive correlation was observed between the child's age and addiction to computer and internet use, showing that older children show a greater degree of addiction (r=0.388, p<0.01).

## Discussion

This study is an attempt to evaluate the addiction of children and teenagers to the use of computer devices and the internet in the current era, a topic that has not been sufficiently studied in Greece, but even in countries that had previous data, the widespread use of digital devices in our everyday life indicates that regular evaluation should be performed. Especially in children and young adolescents the benefits and risks stand in a very sensitive balance. The increasing availability of computers and other digital devices with internet access in homes, as well as the low cost of internet access nowadays, along with wireless accessibility, allows children and adolescents to have online access at any time and place. Many studies support the beneficial use of the internet including early learning, exposure to new ideas, increased opportunities for social contact and support, and easy access to messages and information. On the contrary, there are risks related to problematic internet use that may lead adolescents to aggressive behavior, early sexual activity, drug use, obesity, sleep disorders, eating disorders, depression, suicide, and self-harm [5,16]. Our findings revealed that parents are more sensitive to boys' addiction to internet use since in cases where they had two or more children, they tended to report the internet use for their boys. That may reflect a deeper stereotype that boys are more involved with internet activities and are more addicted to it. In our study, almost half of the parents reported that their children used the internet for two to three hours a day, while the rates of more frequent use were much lower, showing relatively low levels of internet addiction. Studies from international literature agree on the frequency of use, demonstrating an increase in access hours with age [2,8,17]. Following our results, Fam (2018) in a meta-analysis reported a mean prevalence of severe internet addiction in adolescents near 4.1% and that internet addiction in adolescence was positively associated with online chatting, online gaming, and social networking [18,19]. In another recent meta-analysis, a median prevalence rate of 5.5% across all included studies and 2.0% for population-based studies were calculated [20]. Recent studies reinforce the variability of prevalence in different regions of the world [21]. However, we should acknowledge that the addiction prevalence follows the definition of internet addiction as well as the assessment measure used. Therefore, despite the similarities across measures, the comparison of prevalence rates might be challenging.

Our data analysis revealed a tendency for similar internet activities among parents with their children. That can be partly explained by the “role model” influence from parents to their children. Regarding the means of internet access, most parents use smartphones mainly for searching for information, sending emails, and social networking. Similarly, a survey found that adolescents had a higher tendency toward digital device addiction when their parents excessively used them; the findings highlighted the essential role of the parent-child relationship and parental bonding in the formation of internet and smart device addiction [22]. Despite the influence of parent-child relationships (e.g., warmth, conflict, and abuse) and parental guidance on internet use, family attitudes and supervision strategies are also emphasized as key determinants that may contribute to internet addiction [23]. Strict parenting, low self-control, and low self-esteem seem to increase risks for problematic use [9]. There is evidence that patterns of parent screen use are associated with child screen use and child socio-emotional problems, which might lead to problematic internet use [24].

Based on our analysis, nearly six out of 10 children accessed the internet via a smartphone, which is a popular means of connecting to the internet, indicating that the use of mobile phones among children has been very widespread in recent years. A Greek study conducted in 2018 showed that nine out of 10 children had mobile phones and for seven out of 10 children, the mobile phone was the only device to access the internet [8]. Following our findings, a multicenter study of a sample from six different European countries found that a great number of children at the European level (38-60%) spent more than two hours daily on social networking, even during school days [25]. There is a notable increase in the use of smartphones among children and adolescents, even in preschoolers. In a recent review, the use of a smartphone for gaming and social networking rather than communication might increase the risk of addiction. Female adolescents seem to be prone to a higher smartphone addiction risk than male adolescents [9].

Of particular interest is the first age of access to computers and the internet, which started for most children from the ages of seven to nine years old. It is documented that in Greece, most children start to use the internet at the age of seven to eight, and this tends to decrease continuously over time with one in five children beginning to use the internet at a very young and sensitive age of four to six years old (Greek safer internet Center, 2018). Our results add to the current evidence that the prevalence rates of internet addiction have increased over the years and follow the increased rates of internet use nowadays [26]. According to the Pew Research Center (July 2020) in the Report “Parenting Children in the Age of Screens,” more than one-third of parents with a child under 12 stated that their child began interacting with a smartphone before the age of five and nearly one-in-five parents of a child younger than 12 say their child has their smartphone (https://www.pewresearch.org/internet/2020/07/28/childrens-engagement-with-digital-devices-screen-time/).

We found that parents in our sample supervise the internet use of their children based on monitoring the time spent on the internet and computer use and less regarding its content. Moreover, the children tend to ask for more time for internet use based on their parents' reporting. Parents strive to control their child’s internet use and, therefore, the mean daily use is average in comparison to recent studies at the European level [25,27]. Characteristically, we found that three out of 10 children have access for more than four hours daily, increasing the risk of addiction. Therefore, the need for developing and promoting evidence-based guidelines for parents regarding very young children’s engagement with digital technologies and the internet is now clear. Simultaneously, there is a clear need to address the lack of information regarding the risks of internet use for children under nine, for both parents and children [28]. Combined preventive and/or treatment interventions may be beneficial in the early management of internet addiction symptoms [29].

Although boys scored slightly higher than girls (47.53 vs. 44.10) on the Adolescent Computer Addiction scale, this difference was not statistically significant. This is in agreement with previous studies indicating gender differences regarding internet use [2]. Conversely, in other research, the male gender of children and adolescents appeared to be associated with higher rates of internet addiction [18,30-32]. However, the time of internet use differs concerning its content. For example, a recent multicenter study regarding the use of the internet for social networking revealed that girls and older adolescents were more engaged in heavier use compared with boys and younger children [25]. Lee and Kim concluded that younger boys and children who were engaged in internet use for more than an hour daily without adult supervision showed higher odds of internet addiction [27].

Regarding age, increased age appeared to have a significant effect on the likelihood of addiction, a finding that is consistent with that of other studies that show an increase in time of use and addiction with increasing age [2,8,17,33,34]. This is probably because younger children are less independent and more subject to parental control than older children, especially teenagers who are experiencing a time of intense reaction and questioning. Nevertheless, the age of the child's first access to a computer and the internet was not significantly associated with addiction, contrary to the international literature, which demonstrates a strong correlation [2,35,36]. As for the effect of being involved in a sport or other outdoor activities, it did not seem to significantly affect the hours of internet access. Although sports and other outdoor activities have been reported as protective factors against addiction, the usual hours of physical activity per week may not be enough to prevent children from increased access to the internet [37,38]. Leisure boredom increases the probability of internet addiction. Family and outdoor activities along with participative and supportive parental monitoring seem to decrease the risk [39].

According to parental reports, the connection of a computer device to the internet was affecting their willingness to use it. More specifically, half of them did not use it without an internet connection. Consequently, more than 40% of the participants stated that a computer device was available in their child's bedroom, and 60% of these cases with internet access. Regarding this specific parameter, a significant correlation emerged between the availability of a connection in the bedroom and the extensive use of the internet by the children. This sounds reasonable since it makes it easier for children to access the internet without parental control. In a study of 25 countries through child interviews, almost five out of 10 children reported being able to access the internet in their bedroom without restrictions through the "bedroom culture" pattern [2], while a Pan-Hellenic survey showed that the use was made by most children without parental supervision even for primary school children [8]. Adopting the practice of lax or no supervision of network use by parents increases the risk of addiction as it enables them to overuse it. It seems that most parents in Greece do not realize the importance of setting limits and rules concerning internet use for their kids from a very young age, and they lack the required vigilance on this topic [8]. The prevalence of internet addiction is significantly higher among adolescents with a perceived lack of parental supervision and lack of parental connectedness [40]. Strategies for the prevention and treatment of internet addiction in children and young adolescents should focus on improving the quality of parent-adolescent relationships, enhancing social support, and engaging parental supervision with rules and restrictions [41].

In our study, no significant differences were observed between children's increased internet use and parents’ educational level, in line with previous results in Greece [8]. On the contrary, the literature clearly states that the higher educational level of the parents is correlated with greater awareness regarding internet addiction and possible risks [2]. Moreover, Wu et al. (2016) reported that internet addiction was also significantly associated with the mother's and father's education level [42]; however, what we must think about is the distinction between academic knowledge and knowledge regarding safe internet use and addiction.

The study has several limitations that affect the interpretation and generalization of its findings. It was conducted in a convenience sample from a semi-urban area near Athens (Megara). Even though the area was selected to represent the Greek population, the single-center collection does not ensure it. Moreover, the self-report measures and the cross-sectional study design do not allow an in-depth understanding of the phenomenon. Another limitation is that the data was derived through proxy (parental) reports and not directly from children's or adolescents’ reports. Further studies are needed to investigate the addiction phenomenon, considering the digitalization of everyday living, the increased use of digital devices with internet access, and the cultural and behavioral changes that follow this transformation. To better explain the results of this study, follow-up studies including children's assessment and larger samples are required. Nevertheless, there is growing evidence that the design features of studies, such as time frame, geographical region, addiction assessment methods and tools, and sample selection, may greatly influence the assessment of internet addiction.

## Conclusions

The current era is characterized by easy access to communication and information technologies that have increased the use of digital technology and internet use in our daily lives. Most children can go online using a computer device and do so almost twice as much as 10 years ago. They also have internet access at an earlier age than in the past, even from preschool age. Internet access provides children with many opportunities to learn, explore, create, interact, and socialize. Nevertheless, this extensive use of digital information is not free from risks of addiction as well as dysfunctional internet use such as cyberbullying, age-inappropriate content, disinformation, and sexual abuse. Problematic internet use may cause disorders in behavior, sleep, and eating, leading to obesity, early sexual intercourse, drug use, depression, and self-harm.

The trend affects children as well with a decrease in the age of the first engagement with internet access. Parents have no clear direction regarding the benefits and risks involved, and how to effectively support their children’s engagement with the internet in a safe and beneficial way. The results from this study reveal, to some extent, the growing prevalence of internet addiction and the need for public awareness, as well as the importance of prioritizing parents' information and education needs to effectively mediate children's internet use. Hopefully, most of the children did not show a high internet or computer addiction. Certain variables, such as the child's age, the parent’s extensive use of the internet, and internet availability in children’s bedrooms, seem to be associated with higher levels of addiction. More research is needed to address the determinants of internet addiction in children and young adolescents.
